# Supplementary material for: Genomic and epidemiologic characteristics of SARS-CoV-2 persistent infections in California, January 2021 - July 2023
Source: PLoS Pathog. 2025 Nov 10;21(11):e1013365. doi: 10.1371/journal.ppat.1013365 (PMC12614806; doi:10.1371/journal.ppat.1013365)
Supplement: S1 File — (DOCX) [file ppat.1013365.s001.docx]

**Genomic and Epidemiologic Characteristics of SARS-CoV-2 Persistent Infections in California, January 2021 - July 2023**

**Supplemental Guide**

Contents

[Supplementary Guide: CLASSIFYING PERSISTENT INFECTIONS USING GENOMIC CONSIDERATIONS 2](#_Toc202263137)

[UNCERTAINTIES TO CONSIDER WHEN CLASSIFYING CASES AS POTENTIAL INFECTIONS OR REINFECTIONS 2](#_Toc202263138)

[EXAMPLES USING GENOMIC CONSIDERATIONS 4](#_Toc202263139)

[PERSISTENT INFECTION: CASE 17 4](#_Toc202263140)

[PERSISTENT INFECTION: CASE 52 6](#_Toc202263141)

[REINFECTION 9](#_Toc202263142)

[PERSISTENT INFECTION: CASE 30 12](#_Toc202263143)

[PERSISTENT INFECTION: CASE 22 16](#_Toc202263144)

## Supplementary Guide: CLASSIFYING PERSISTENT INFECTIONS USING GENOMIC CONSIDERATIONS

The purpose of this manual is to provide additional detail on the genomic considerations used to determine whether SARS-CoV-2 sequences collected from the same individual comprise a persistent infection or reinfection.

These considerations are not without subjectivity and may involve judgement calls from bioinformaticians and genomic epidemiologists with SARS-CoV-2 subject matter expertise.

### UNCERTAINTIES TO CONSIDER WHEN CLASSIFYING CASES AS POTENTIAL INFECTIONS OR REINFECTIONS

**ARE THE GENOME ASSEMBLIES SUFFICIENT QUALITY?**

ARE THE ASSEMBLIES WITHIN A FEW MUTATIONS OF DIVERGENCE?

ARE CHANGES IN ALLELE FREQUENCIES INDICATIVE OF WITHIN-HOST EVOLUTION?

ARE GLOBAL CONTEXTUAL ASSEMBLIES CLOSELY RELATED TO LATER ASSEMBLY?

- A genome assembly can have a large number of ambiguous sites (Ns) and still be categorized as a persistent infection, so setting specific QC thresholds for genome assemblies is a challenge
- For persistent infections with specimens collected further apart, more Ns would be expected, as Intra-host evolution could result in mixed alleles whereby there wouldn’t be a “consensus” for the nucleotide at those positions in the genome assembly
- When genome assemblies are lower quality, read data needs to be further investigated

ARE THE GENOME ASSEMBLIES SUFFICIENT QUALITY?

**ARE THE ASSEMBLIES WITHIN A FEW MUTATIONS OF DIVERGENCE?**

ARE CHANGES IN ALLELE FREQUENCIES INDICATIVE OF WITHIN-HOST EVOLUTION?

ARE GLOBAL CONTEXTUAL ASSEMBLIES CLOSELY RELATED TO LATER ASSEMBLY?

- Genome assemblies that are within a few mutations of divergence are very likely to be persistent infections, as the odds of being infected with a nearly identical SARS-CoV-2 virus twice are extremely low
- For highly divergent genome assemblies, if they share a common ancestor on a phylogenetic tree, reinfection is most likely; if one appears ancestral to the other, persistent infection is more likely
- The window of time between collection dates needs to be considered, as more divergence would be expected over longer periods of time
- Divergence is difficult to measure using phylogenetic placement when a genome assembly is poor quality, so further investigation is often needed to identify the sites that differ between the genomes

ARE THE GENOME ASSEMBLIES SUFFICIENT QUALITY?

ARE THE ASSEMBLIES WITHIN A FEW MUTATIONS OF DIVERGENCE?

ARE CHANGES IN ALLELE FREQUENCIES INDICATIVE OF WITHIN-HOST EVOLUTION?

**ARE GLOBAL CONTEXTUAL ASSEMBLIES CLOSELY RELATED TO LATER ASSEMBLY?**

- If there are many contextual genome assemblies closely related to the later genome, reinfection is more likely, but this is only relevant if the time window between collection dates is long enough for divergence to occur
- As collection rates have been dropping since early 2023, it becomes more and more difficult to evaluate the weight of contextual sequences as evidence for reinfection

ARE THE GENOME ASSEMBLIES SUFFICIENT QUALITY?

ARE THE ASSEMBLIES WITHIN A FEW MUTATIONS OF DIVERGENCE?

**ARE CHANGES IN ALLELE FREQUENCIES INDICATIVE OF WITHIN-HOST EVOLUTION?**

ARE GLOBAL CONTEXTUAL ASSEMBLIES CLOSELY RELATED TO LATER ASSEMBLY?

- This only needs to be considered if the genome assemblies were not high enough quality to have confidence in the phylogenetic placement
- The reason for the large number of Ns in genome assemblies could be:
  - **Contamination**: if this is occurring, there will be many mixed sites throughout the entire genome in the variant caller output, and likely phasing of mutations when visualizing mapped reads in IGV (phasing is more evident for sequences with recent collection dates, as there is more diversity relative to Wuhan-1 over time)
    - If contamination or co-infection is suspected, the case should be excluded from categorization
  - **Intra-host evolution:** if this is occurring, there will be some mixed sites in the variant caller output, with little or no phasing of mutations unless viral sub-populations are suspected
    - If mixed sites are shared between sequences of the same case, persistent infection is likely
    - If there is evidence of a mutation gradually arising to fixation or reverting, persistent infection is likely
    - The longer the time window between collection dates, the higher the likelihood of Intra-host evolution and potential emergence of viral subpopulations
    - If unable to distinguish Intra-host evolution from contamination, the case should be excluded from categorization

### EXAMPLES USING GENOMIC CONSIDERATIONS

Case numbers can be found in Supplemental Table 1.

#### PERSISTENT INFECTION: CASE 17

ARE THE GENOME ASSEMBLIES SUFFICIENT QUALITY?

ARE THE ASSEMBLIES WITHIN A FEW MUTATIONS OF DIVERGENCE?

ARE CHANGES IN ALLELE FREQUENCIES INDICATIVE OF WITHIN-HOST EVOLUTION?

ARE GLOBAL CONTEXTUAL ASSEMBLIES CLOSELY RELATED TO LATER ASSEMBLY?

**YES**

**YES**

**YES**

**YES**

- Both assemblies have high percent reference coverage (Table 1)
- There is a large discrepancy in mean assembly depth, but the lower value for 6000002412 is still almost 700x
- Classification based on genomes alone will likely suffice, unless oddities in phylogeny are identified
- On the UShER global tree, the first genome appears ancestral to the later genome, a likely indicator of ongoing evolution (Figure 1)
- On the UShER subtree, there are no global contextual genomes closely related to the later genome (Fig 2)
- The first genome, located on the same subtree, is also 4 mutations divergent from global contextual genomes, indicating that this case may have been infected for a while before getting testing for COVID-19
- Unnecessary to evaluate given high quality of sequence data

**This case was ruled as a persistent infection given the high-quality sequence data which demonstrated a clear pattern of ongoing evolution within the host.**

Table 1. Collection dates and assembly quality metrics for Case 17

| **Genome** | **GISAID Accession** | **Collection Date** | **Percent Reference Coverage** | **Mean Assembly Sequencing Depth** |
| --- | --- | --- | --- | --- |
| 6000002412 | EPI_ISL_9756334 | 2021-09-06 | 96.6% | 699.3x |
| ASC210462914 | EPI_ISL_6163665 | 2021-10-13 | 99.6% | 5366.7x |


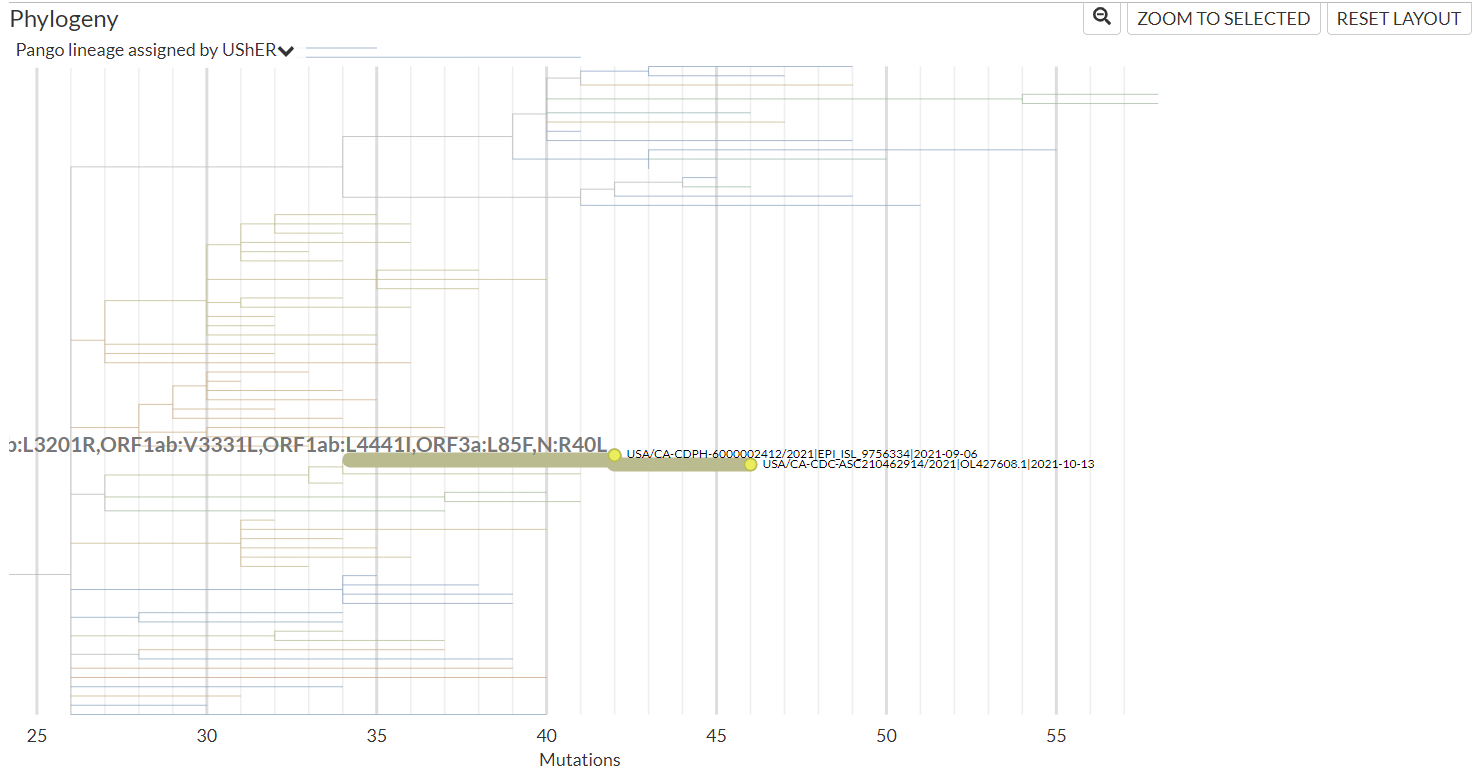


Figure 1. UShER global tree demonstrating the later genome diverging from the first genome, indicating ongoing evolution


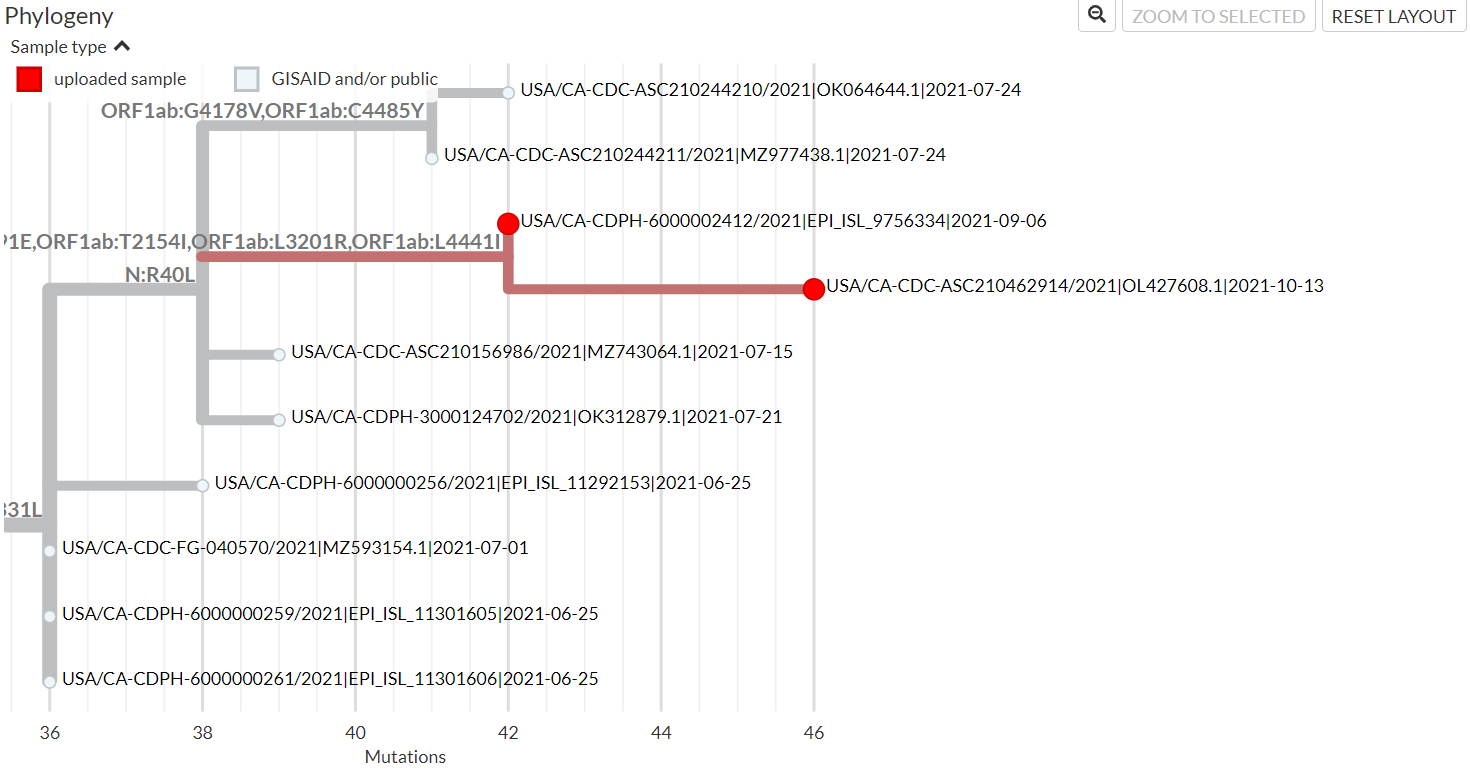


Figure 2. UShER subtree showing that there are no global contextual genomes closely related to later genome

#### PERSISTENT INFECTION: CASE 52

ARE THE GENOME ASSEMBLIES SUFFICIENT QUALITY?

ARE THE ASSEMBLIES WITHIN A FEW MUTATIONS OF DIVERGENCE?

ARE CHANGES IN ALLELE FREQUENCIES INDICATIVE OF WITHIN-HOST EVOLUTION?

ARE GLOBAL CONTEXTUAL ASSEMBLIES CLOSELY RELATED TO LATER ASSEMBLY?

**YES**

**YES**

**YES**

**YES**

- Both assemblies have good percent reference coverage (Table 2)
- There is a large discrepancy in mean assembly depth, but the lower value for 500103333 is still >700x
- Classification based on genomes alone will likely suffice, unless oddities in phylogeny are identified
- On the UShER global tree, the assemblies are identical (Fig 3)
- On the UShER subtree, there are many assemblies identical to the later genome (Fig 4)
- While in some instances that might suggest reinfection, given the short time window between collection dates in this case and compared to global contextual genomes, we would not expect much divergence to have occurred anyway
- Unnecessary to evaluate given high quality of sequence data

**This case was ruled as a persistent infection considering the extremely low likelihood of an individual being infected with two, nearly identical SARS-CoV-2 viruses approximately 5 weeks apart.**

Table 2. Collection dates and assembly quality metrics for Case 52

| **Genome** | **GISAID Accession** | **Collection Date** | **Percent Reference Coverage** | **Mean Assembly Sequencing Depth** |
| --- | --- | --- | --- | --- |
| 500103333 | EPI_ISL_17291401 | 2023-01-28 | 92.1% | 718.8x |
| 500107770 | EPI_ISL_17529665 | 2023-03-04 | 99.3% | 12658.3x |


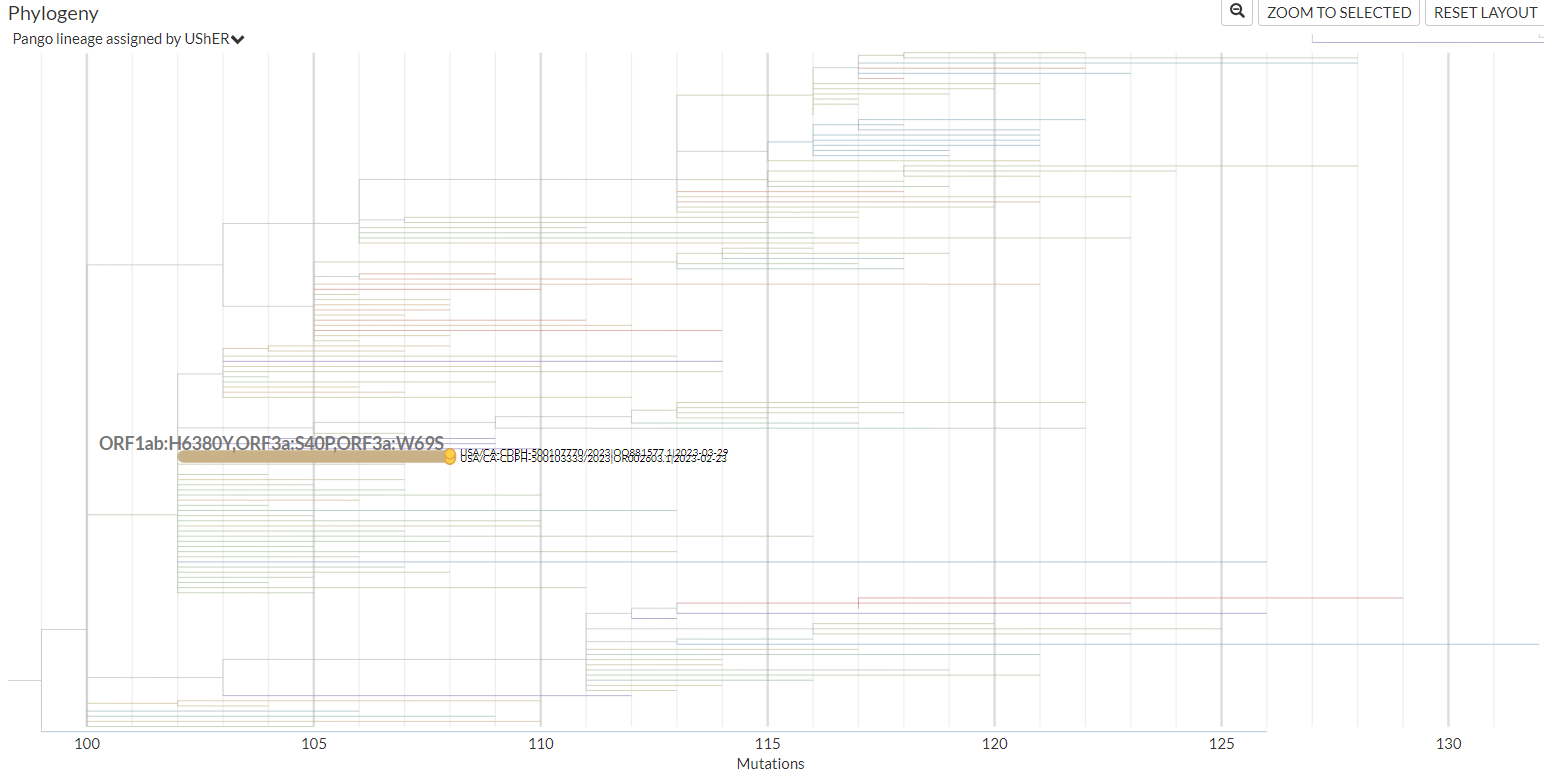


Figure 3. UShER global tree demonstrating identical genome assemblies


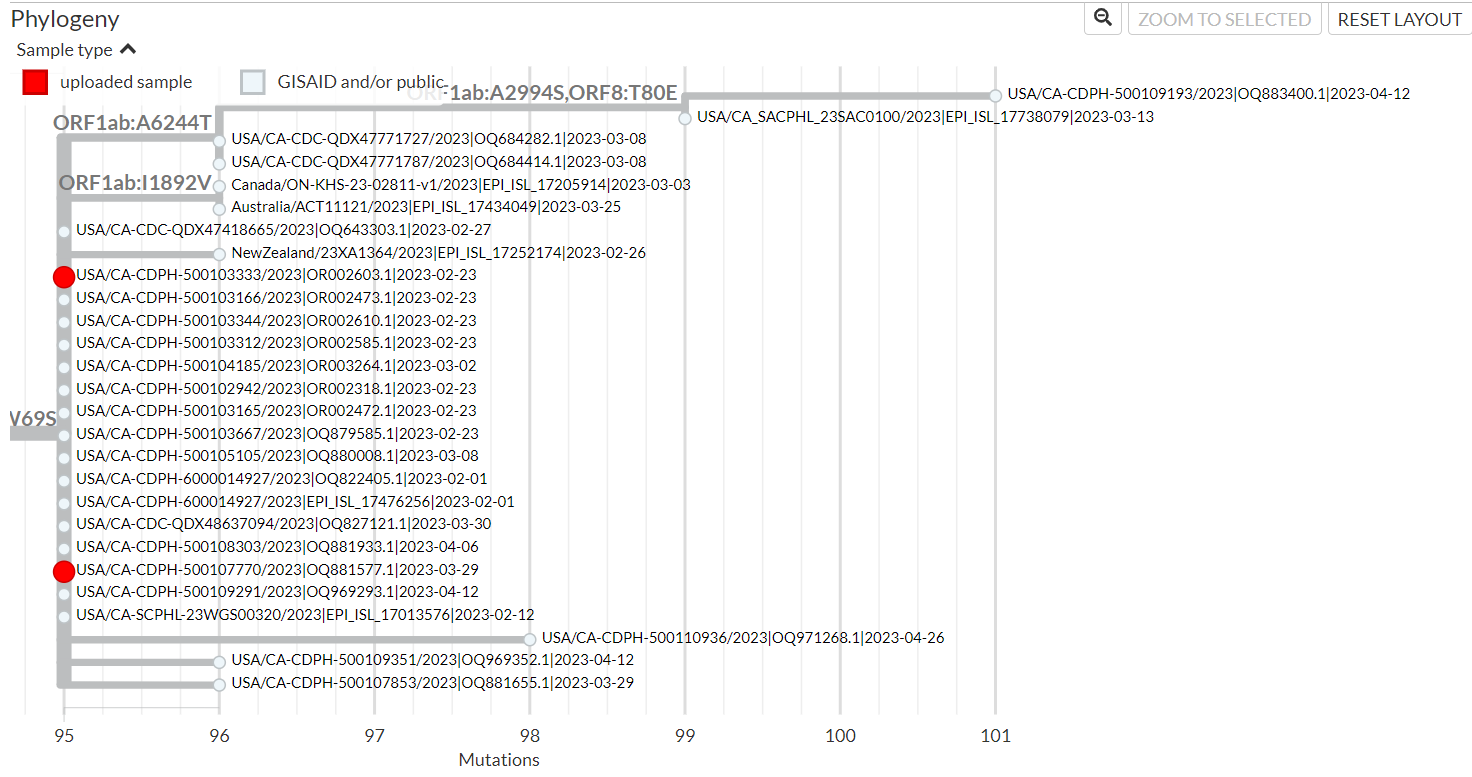


Figure 4. UShER subtree demonstrating many global contextual genome assemblies identical to those in Case 52

#### REINFECTION

ARE THE GENOME ASSEMBLIES SUFFICIENT QUALITY?

ARE THE ASSEMBLIES WITHIN A FEW MUTATIONS OF DIVERGENCE?

ARE CHANGES IN ALLELE FREQUENCIES INDICATIVE OF WITHIN-HOST EVOLUTION?

ARE GLOBAL CONTEXTUAL ASSEMBLIES CLOSELY RELATED TO LATER ASSEMBLY?

**YES**

**YES**

**YES**

**YES**

- Both assemblies have high percent reference coverage (Table 3)
- There is a large discrepancy in mean assembly depth, but the lower value for FS25441776 is still 544x
- Classification based on genomes alone will likely suffice, unless oddities in phylogeny are identified
- On the UShER global tree, the genome assemblies are very divergent and share a common ancestor (Fig 5)
- The genome assemblies originate from specimens collected less than a month apart, which would not be enough time for this divergence to have resulted from intrahost evolution
- On the UShER subtree, there are several global contextual genomes identical to the later genome (Fig 6)
- Unnecessary to evaluate given high quality of sequence data

**This case was ruled as a reinfection since the high-quality genome assemblies were much more divergent from one another than would be expected for a persistent infection in this time window, and there were global contextual genomes identical to the later genome.**

Table 3. Collection dates and assembly quality metrics for example reinfection

| **Genome** | **GISAID Accession** | **Collection Date** | **Percent Reference Coverage** | **Mean Assembly Sequencing Depth** |
| --- | --- | --- | --- | --- |
| FS25441776 | EPI_ISL_4868988 | 2021-07-22 | 93.9% | 544.6x |
| FF08510938 | EPI_ISL_4870010 | 2021-08-13 | 99.9% | 5834.8x |


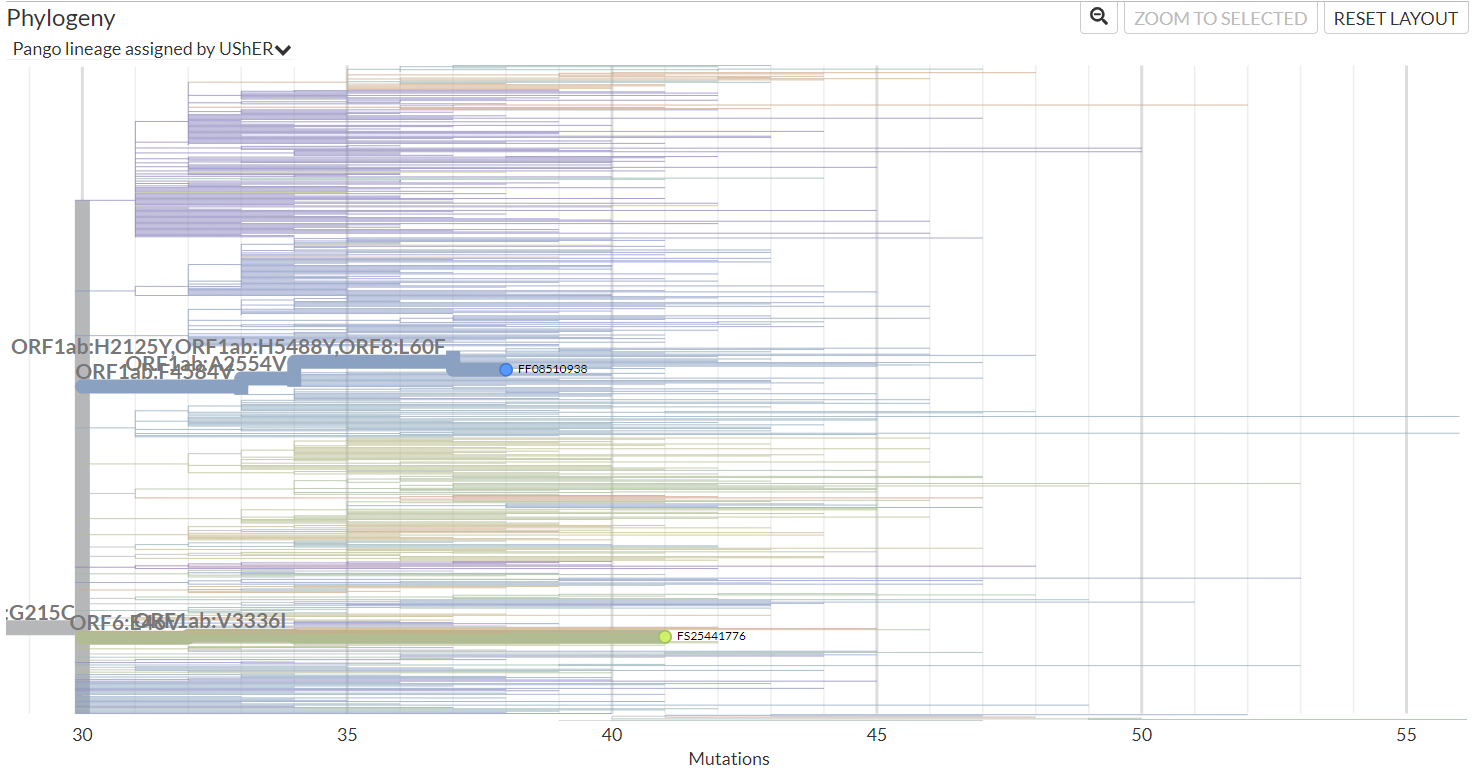


Figure 5. UShER global tree demonstrating very divergent genome assemblies that share a common ancestor


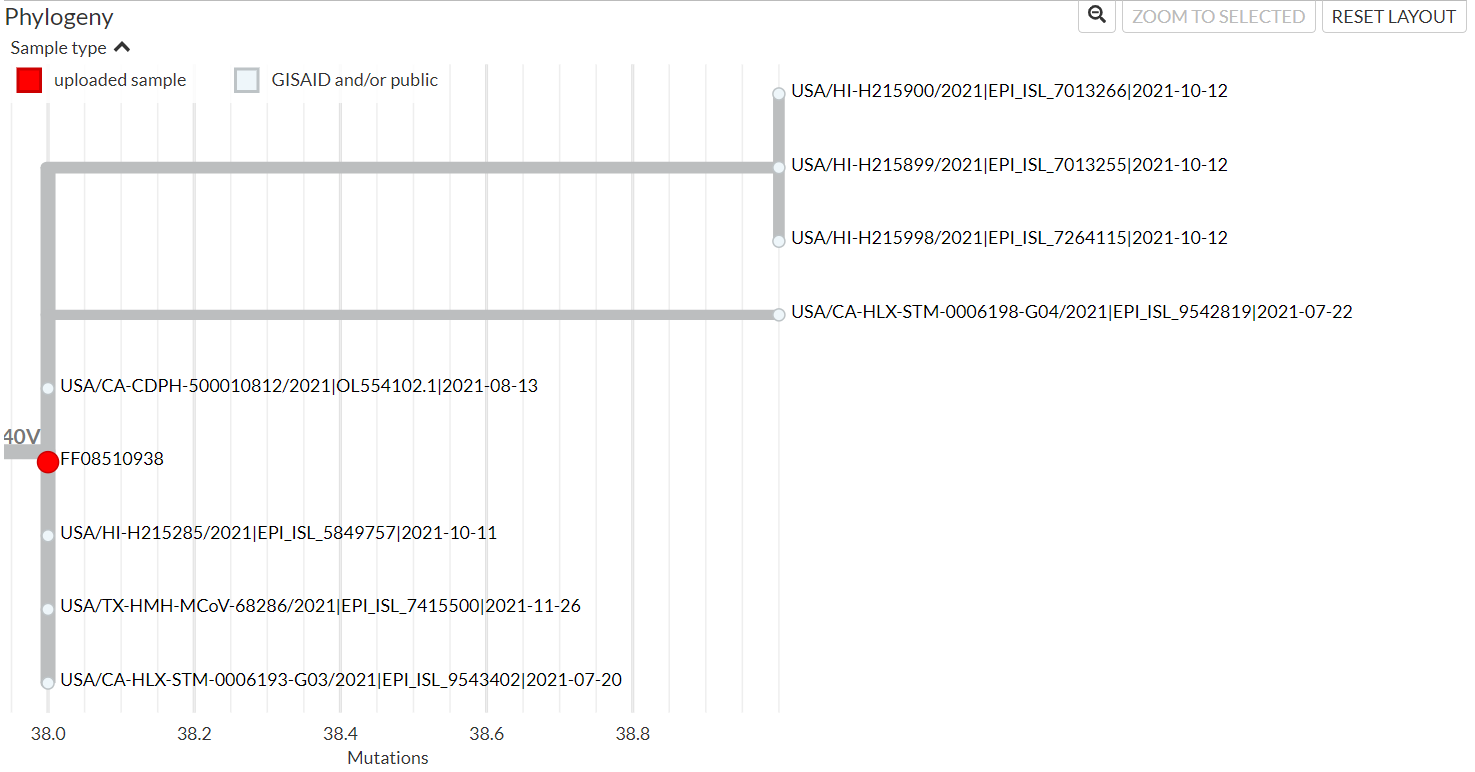


Figure 6. UShER subtree showing many global contextual genomes identical or closely related to later genome

#### PERSISTENT INFECTION: CASE 30

ARE THE GENOME ASSEMBLIES SUFFICIENT QUALITY?

ARE THE ASSEMBLIES WITHIN A FEW MUTATIONS OF DIVERGENCE?

ARE CHANGES IN ALLELE FREQUENCIES INDICATIVE OF WITHIN-HOST EVOLUTION?

ARE GLOBAL CONTEXTUAL ASSEMBLIES CLOSELY RELATED TO LATER ASSEMBLY?

**NO**

**NO**

**YES**

**YES**

- One assembly (ASC210756442) is missing ~20% of the genome, and another (ASC210553265) is missing ~9% (Table 4)
- All genomes have strings of Ns within the Spike protein which should contain mutations (Fig 7)
- Phylogenetic analysis will not be sufficient for determining whether this is a persistent infection
- While two genomes (ASC210553265 and ASC210818864) appear 3 mutations divergent, the other(ASC210756442) genome with the later collection date is at least 10 mutations divergent from those (Fig 8)
- The later genome has a collection date less than a month following the previously collected genome, so that level of divergence is unexpected and likely due to the missing regions within the assembly
- On the UShER subtree, there are several global contextual genomes identical to the later genome (Fig 9)
- It is unclear whether this is due to missing regions within the assembly, not enough time between collection dates, or a combination a both
- The Ns in ASC210756442 were due to poor sequencing depth (Fig 10), not contamination (Fig 11)
- There is a shared minor allele between the first and second sequence at 15521bp, which is fixed in the last sequence, demonstrating evolution over time

**This case was ruled as a persistent infection given that there were 3 BA.1 sequences from the same individual collected within a 30-day time window, and there was a shared minor allele in the first two sequences that arose to fixation in the third sequence.**

Table 4. Collection dates and assembly quality metrics for Case 30

| **Genome** | **GISAID Accession** | **SRA Accession** | **Collection Date** | **Percent Reference Coverage** | **Mean Assembly Sequencing Depth** |
| --- | --- | --- | --- | --- | --- |
| ASC210553265 | EPI_ISL_10373187 | SRR20902781 | 2022-02-11 | 91.2% | 3817.1x |
| ASC210818864 | EPI_ISL_10491670 | SRR20902781 | 2022-02-16 | 97.4% | 54381.0x |
| ASC210756442 | Not in GISAID | SRR18775147 | 2022-03-04 | 79.4% | 6825.3x |


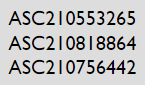

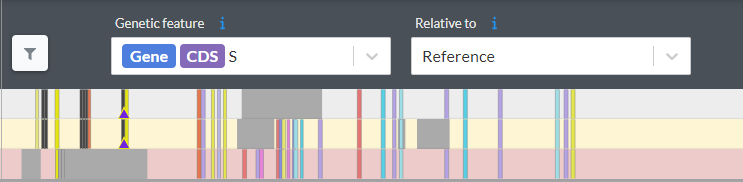


Figure 7. Spike protein amino acid changes (colors) and ambiguous sites (gray), demonstrating many ambiguous sites as well as identical amino acid mutational profiles outside of ambiguous sites


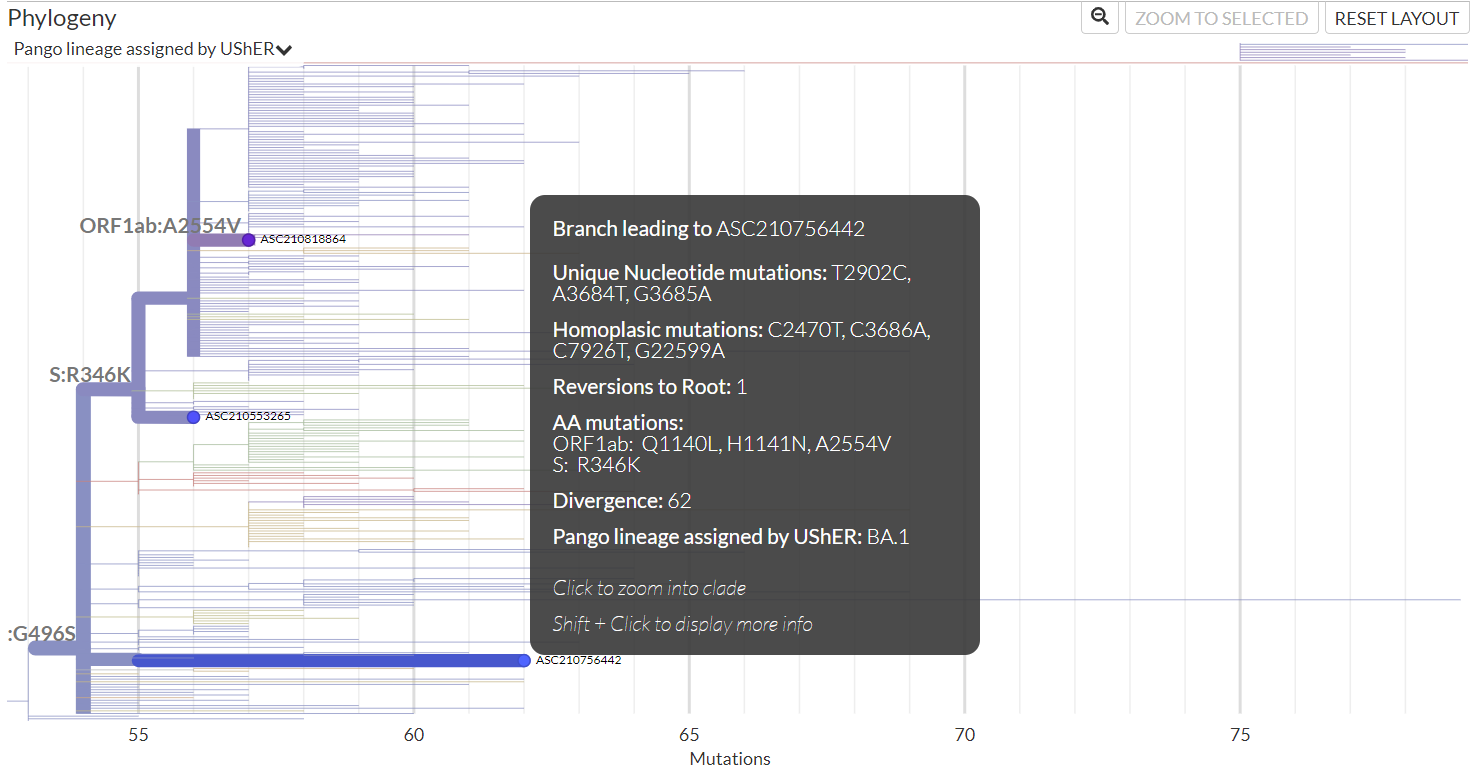


Figure 8. UShER global tree demonstrating placement of 3 genome assemblies on different branches, but shared mutations (such as S:R346K & ORF1ab:A2554V) across those branches, which may indicate that poor sequence quality is impeding phylogenetic placement


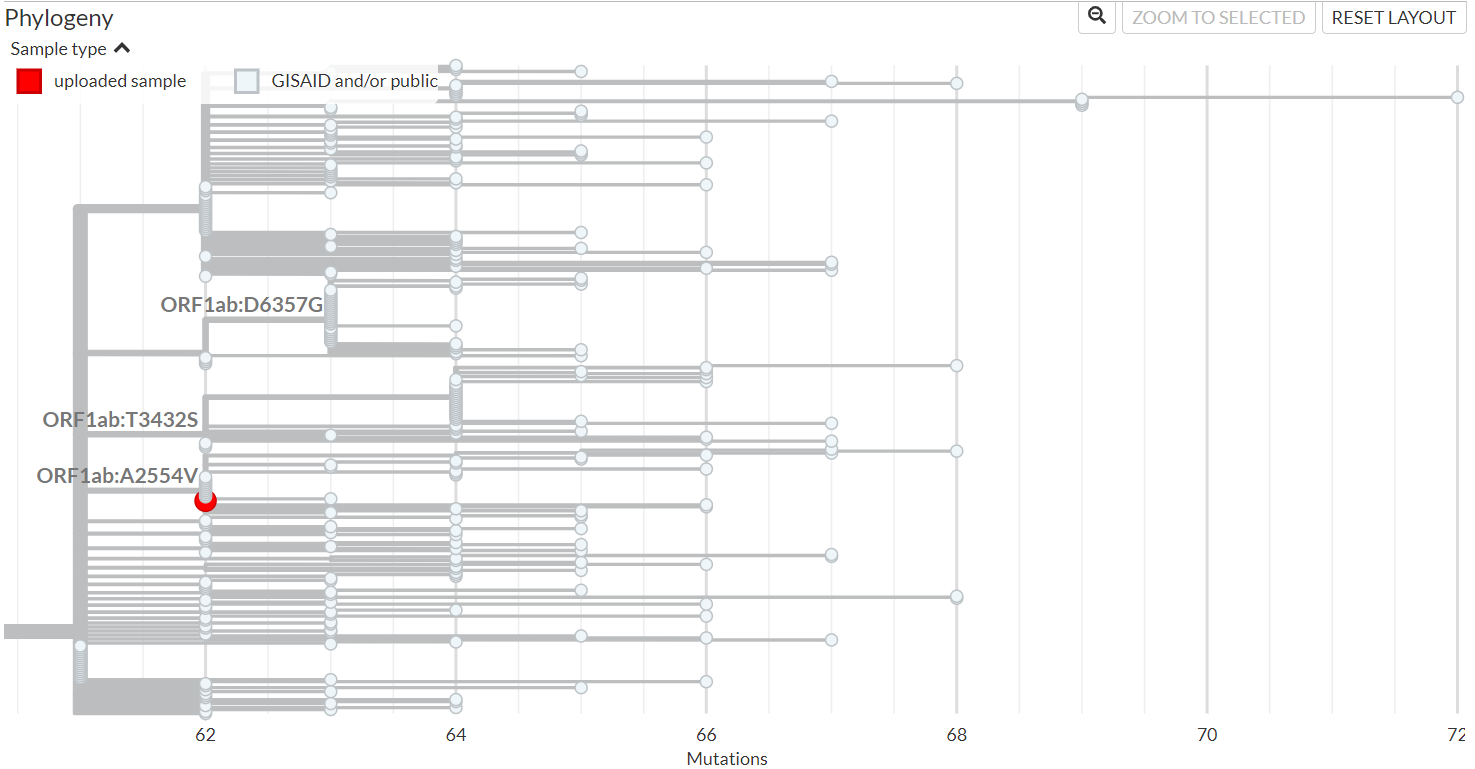


Figure 9. UShER subtree showing the later genome appearing identical to global contextual assemblies, which may or may not be due to the large number of ambiguous sites


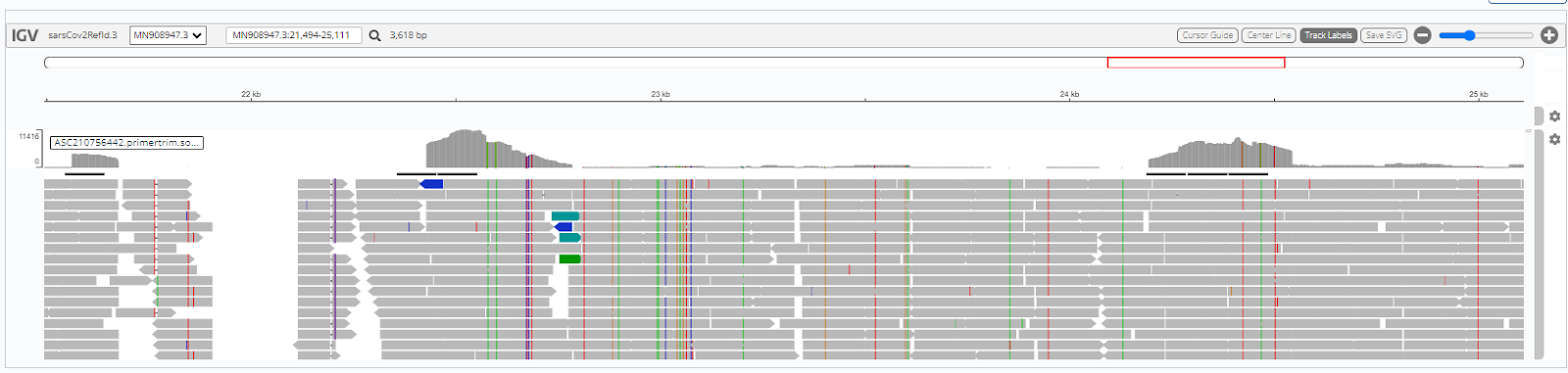


Figure 10. Read mapping to Wuhan-1 reference genome visualized in IGV, zoomed into Spike gene to demonstrate vast differences in sequencing depth


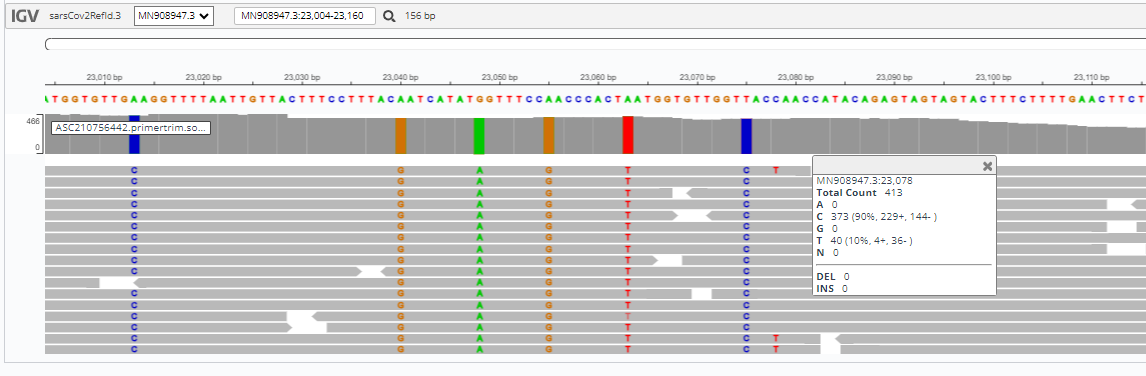


Figure 11. Read mapping to Wuhan-1 reference genome visualized in IGV, zoomed into mutational hotspot within spike to show a mixed site (23,078bp) and lack of phasing


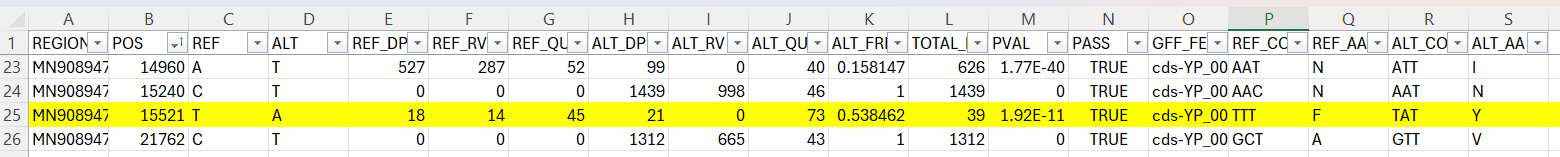


Figure 12. Alternative nucleotide position 15521 in ASC210553265 AT 54% frequency


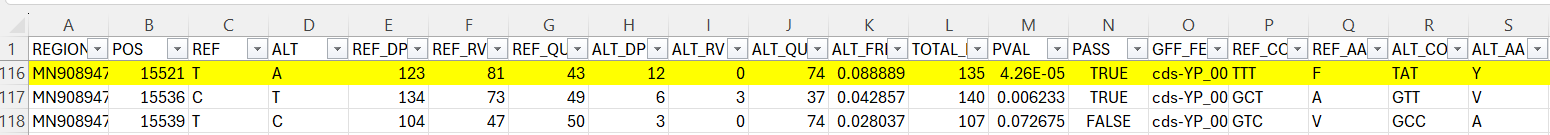


Figure 13. Alternative nucleotide position 15521 in ASC210818864 at 9% frequency


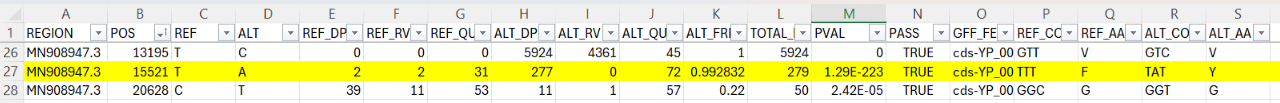


Figure 14. Alternative nucleotide position 15521 in ASC210756442 at 99% frequency

#### PERSISTENT INFECTION: CASE 22

ARE THE GENOME ASSEMBLIES SUFFICIENT QUALITY?

ARE THE ASSEMBLIES WITHIN A FEW MUTATIONS OF DIVERGENCE?

ARE CHANGES IN ALLELE FREQUENCIES INDICATIVE OF WITHIN-HOST EVOLUTION?

ARE GLOBAL CONTEXTUAL ASSEMBLIES CLOSELY RELATED TO LATER ASSEMBLY?

**NO**

**YES**

**YES**

**YES**

- The second genome assembly (3000270125) is missing ~20% of the genome (Table 5)
- The missing regions in the second genome overlap with sites that should contain mutations shared with the first genome (Fig 12)
- Phylogenetic analysis will not be sufficient for determining whether this is a persistent infection
- The genomes are two mutations divergent according to UShER, but this wouldn’t capture any mutations within the missing regions of the second assembly (Fig 16)
- On the UShER subtree, there are several global contextual genomes within a few mutations of divergence of the second genome (Fig 17)
- While both genomes fall on this subtree, the second genome appears ancestral to the first, likely due to missing regions
- The Ns in 3000270125 were due to poor sequencing depth, not contamination (Fig 18)
- There are shared mixed sites at 24794 bp and 28271bp in both 500023760  and 3000270125 (Fig 20-21)

**This case was ruled as a persistent infection given that there were two sequences from the same individual, collected just over a month apart, that shared multiple mixed nucleotide sites.**

Table 5. Collection dates and assembly quality metrics for Case 22

| **Genome** | **Accession** | **Collection Date** | **Percent Reference Coverage** | **Mean Assembly Sequencing Depth** |
| --- | --- | --- | --- | --- |
| 500023760 | SRR17101732 | 2021-10-04 | 99.1% | 4799.6x |
| 3000270125 | SRR17100650 | 2021-11-09 | 80.8% | 1408.8x |


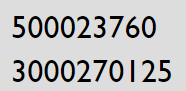

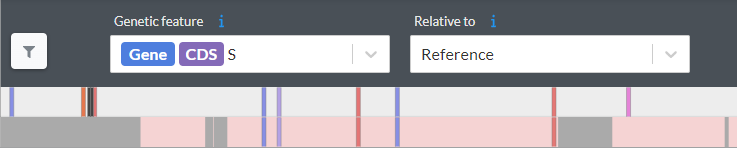


Figure 15. Spike protein amino acid changes (colors) and ambiguous sites (gray), demonstrating many ambiguous sites in the second genome


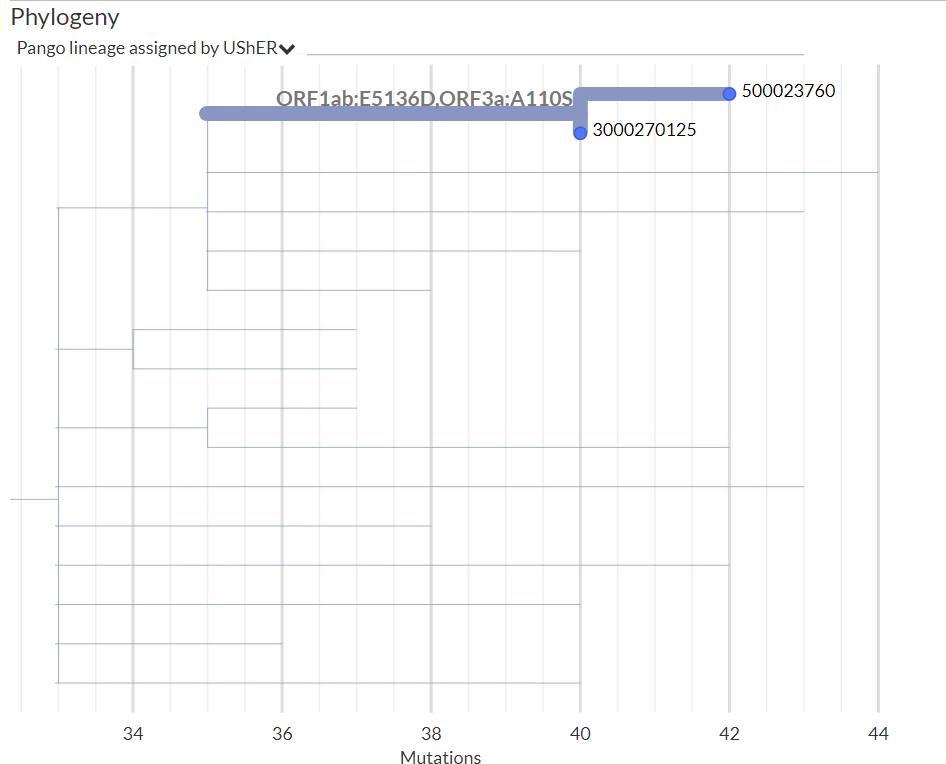


Figure 16. UShER global tree demonstrating the consensus genome assemblies are 2 mutations divergent


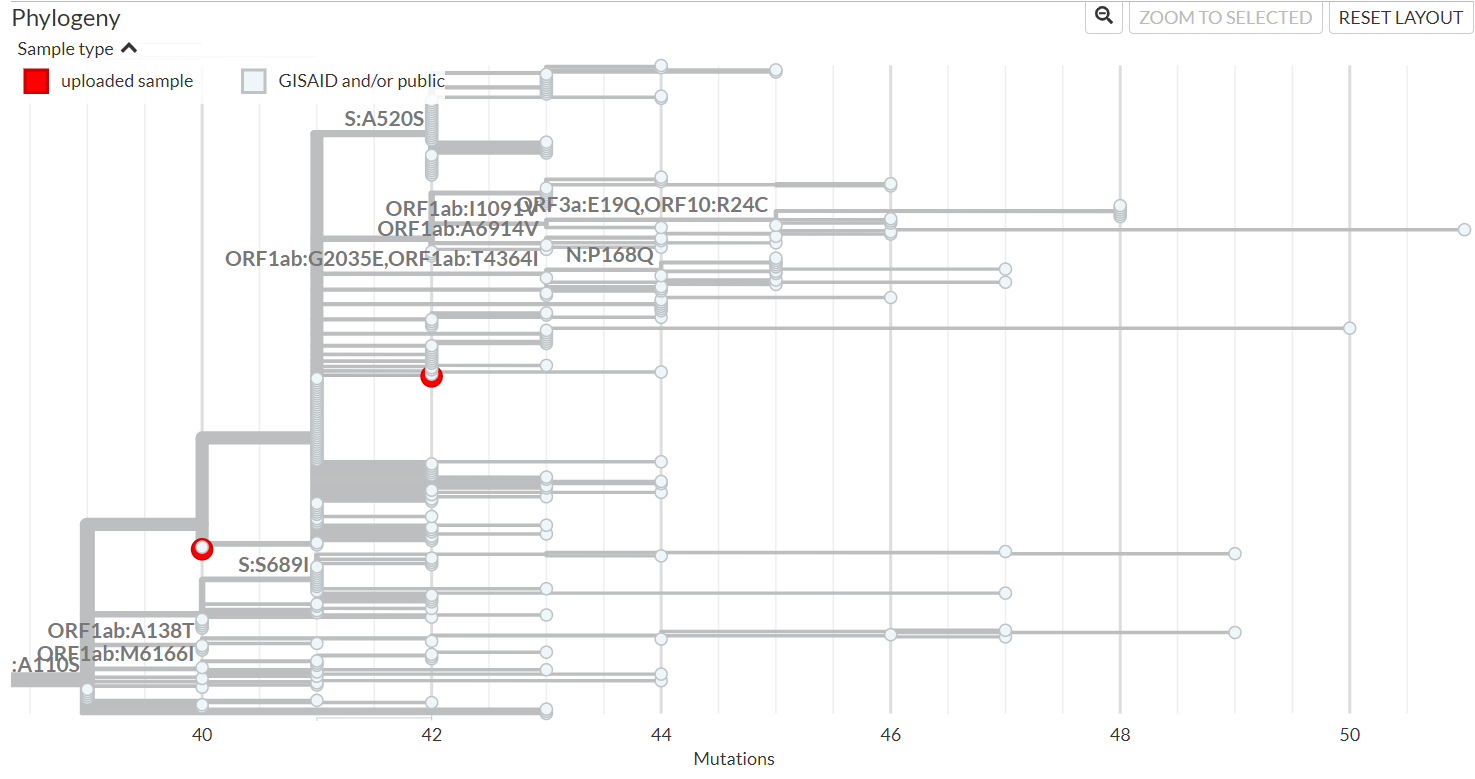


Figure 17. UShER subtree showing that there are many genomes within a few mutations of divergence to the second genome


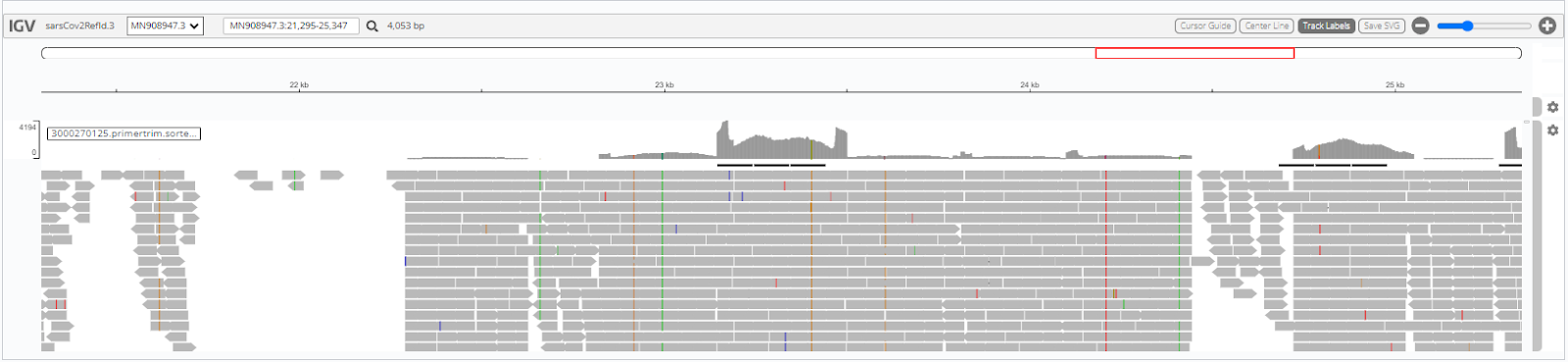


Figure 18. Read mapping of 3000270125 to Wuhan-1 reference genome visualized in IGV, zoomed into region in ORF1a to demonstrate major differences in sequencing depth


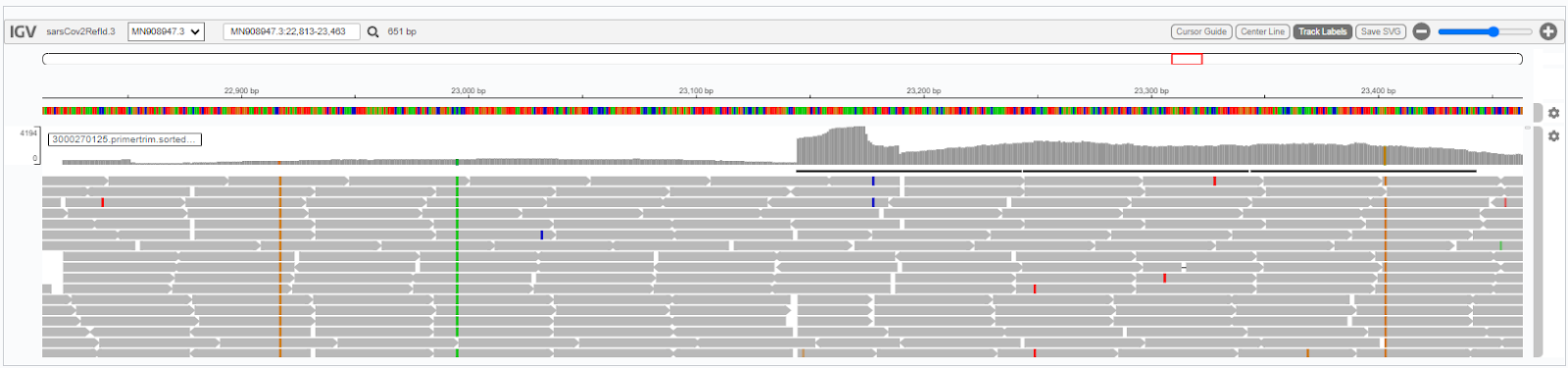


Figure 19. Read mapping of 3000270125 to Wuhan-1 reference genome visualized in IGV, zoomed into region of Spike with several fixed mutations and multiple minor alleles with no phasing


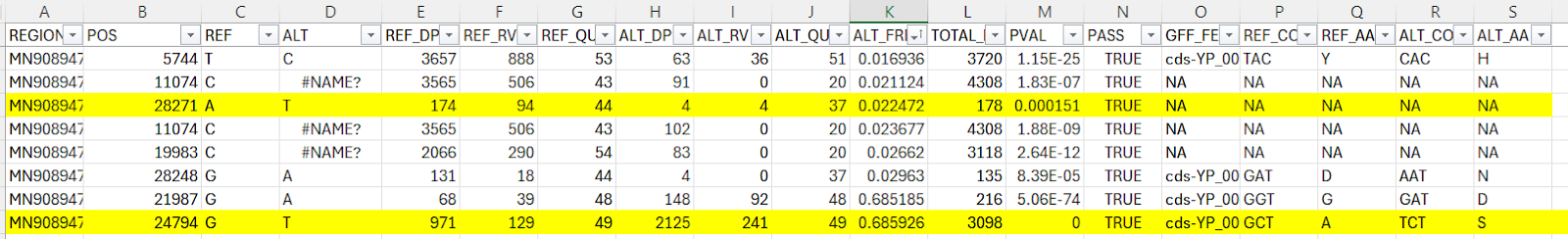


Figure 20. Alternative nucleotide positions 24794 and 28271 in 500023760


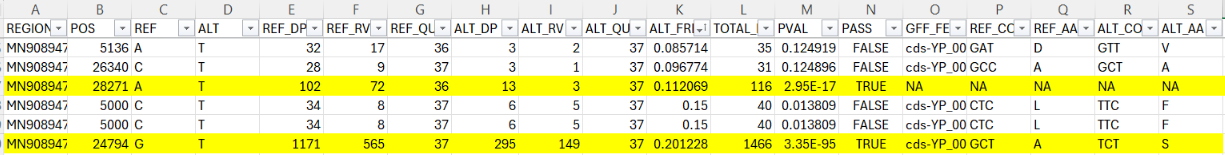


Figure 21. Alternative nucleotide positions 24794 and 28271 in 3000270125
