# Supplementary figures and images for: Genomic and epidemiologic characteristics of SARS-CoV-2 persistent infections in California, January 2021 - July 2023
Source: PLoS Pathog. 2025 Nov 10;21(11):e1013365. doi: 10.1371/journal.ppat.1013365 (PMC12614806; doi:10.1371/journal.ppat.1013365)

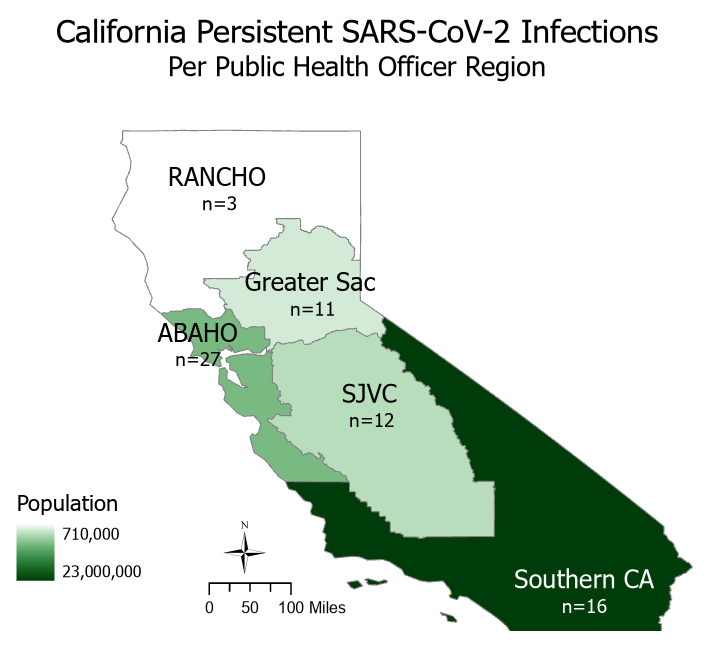

Supplement: S1 Fig — N is the count of persistent SARS-CoV-2 infections identified in this paper. PHO regions are colored according to 2023 population size (worldpopulationreview.com accessed 4 March 2024). Map was created using ArcGIS Pro (version 3.0.0). The original County lines were downloaded from https://catalog.data.gov/dataset/ca-geographic-boundaries. This is an open access dataset in the public domain and no license information is provided. (PNG) [file ppat.1013365.s002.png]
