# Supplementary material for: Genomic and epidemiologic characteristics of SARS-CoV-2 persistent infections in California, January 2021 - July 2023
Source: PLoS Pathog. 2025 Nov 10;21(11):e1013365. doi: 10.1371/journal.ppat.1013365 (PMC12614806; doi:10.1371/journal.ppat.1013365)
Supplement: Attachment — Submitted filename: Response to reviewers PPATHOGENS-D-25-01692 FINAL.docx [file ppat.1013365.s008.docx]

**Reviewer 2 comment #1:**

*“Analyses done in this study have a critical problem due to the criteria for detecting persistent SARS-CoV-2 infections. A 2024 paper by Machkovech et al, which is the reference 1 in the manuscript, defined the persistent SARS-CoV-2 infection as follows: At least one of the following for at least 30 days after symptom onset: 1) isolation of replication-competent virus, 2) accumulation of viral genetic changes over time, 3) cycle threshold values 30 or lower, for 30 days or longer or antigen test positivity, 4) detectable subgenomic RNA, 5) ongoing symptoms.

However, the selection criteria for persistent SARS-CoV-2 shown in Figure 1 in this manuscript are looser than Machkovech’s definition. Even if two sequences were isolated from a patient with more than 21 days' difference, this did not necessarily mean the second sequences were collected at least 30 days after symptom onset. The authors should have described the reasons why they set the criteria for detecting persistent SARS-CoV-2 infections as shown in Figure 1.

The characteristics of the SARS-CoV-2 infections described in this manuscript may not be those of persistent SARS-CoV-2 infections but those of patients from whom viral sequences were collected more than 21 days apart.

The reviewer is wondering why the authors did not detect persistent SARS-CoV-2 infections by comparing the date of symptom onset and the date of sample collection for sequencing. This will be a more straightforward criterion to detect persistent SARS-CoV-2 infections using epidemiological data and sequencing data than those used in this manuscript.*

**Authors’ Response:**

We thank the reviewer for the opportunity to clarify our methods. Based on our literature review, there was no consensus as to the length of SARS-CoV-2 infection before considering it persistent. One recent study used a time frame as short as 14 days (Zhang et al, 2025), and the article the reviewer highlighted used 30 days. One of the purposes of this study was to illustrate what can be elucidated from the genomic data and limited epidemiological data that public health entities, such as laboratories, can access. Symptom onset date and clearance date are frequently missing from our surveillance epidemiological data, and only specimen collection dates are consistently available for each sequence. Hence, we used a minimum difference of 21 days between specimen collection dates as the only metric available across the entire dataset. We acknowledge this as a limitation in our results by stating “the number of days reflects the duration between specimen collection dates, which may or may not reflect the actual duration of infection.”

We have taken this opportunity to expand upon our definition of persistent infection and thought process behind selecting 21 days. The introduction now contains information on a lack of uniform definition for persistent infection (lines 86-90), the first paragraph of the results now contains the 21-day time frame (lines 116-118), and the methods section now has additional detail on the 21-day time frame (lines 463-467).

**Reviewer 2 comment #2:**

*The analysis of positive selection in this manuscript also has a problem. Positively selected codons should be detected by comparing the non-synonymous and synonymous substitution rates. See, for instance, a research of influenza A viruses by Bush et al.”*

**Authors’ Response:**

We appreciate the feedback from Reviewer #3 regarding the use of the term positive selection and given that we did not compare non-synonymous and synonymous substitution rates, we have accordingly omitted the term “positive selection” in this manuscript and modified our language to refer only to nonsynonymous mutational enrichment (Line 53-55, 222 [Fig 3 caption], 241, 321-322, 329-331). This report is intended to provide a high-level overview of the genomic and epidemiologic trends in persistent infections, and we leave more in depth analyses into the evolutionary underpinnings of this mutation enrichment, such as dN/dS ratios and nucleotide diversity (*π*), for future study.

**Reviewer 3 comments #1:**

*Line 63: A comma should be added after i.e., and the author is advised to modify it*

*Retrospective analysis revealed 69 persistent infection cases in California*

*Line 116: For smaller numbers, usually numbers less than 10, it is better to spell them in English, and they are also formal. I recommend that authors review and standardize digital writing throughout the manuscript.*

*I suggest that the author enhance the clarity of Figures to improve the visual quality of the presentation.*

**Authors’ Response:**

We thank Reviewer 3 for the suggestions above and appreciate the opportunity to address these issues. We inserted a comma after “i.e.” on line 63 and have also updated text throughout the manuscript such that all numbers less than 10 are spelled out.

With respect to enhancing Figures for visual clarity, we reviewed all figures to assess visual clarity. We modified language to better describe iSNV frequencies (lines 247-249) attendant to Figure 3b. We also removed Figure 4c from the manuscript, which was admittedly vague, and added text that better describes and identifies phasing of reversions and mutations (lines 355-359).

**Reviewer 3 comment #2:**

*Medical records indicate presence of comorbidities in subset of persistent infection cases*

*Patients with cancer should be analyzed for co-infection in cases with persistent infection, whether the mutation of positive selection of immune escape is due to cancer, and whether the therapeutic agents used in each case have an effect on mutation accumulation.*

**Authors’ Response:**

Thank you for this comment. Although we agree that in depth analysis and monitoring of cancer patients with COVID, as suggested, would be quite informative from clinical as well as virologic perspectives, specifically for identifying mutation enrichment and correlation with therapeutics used in these patients, that type of investigation is beyond the scope of our abilities and is better suited for medical investigators that can track COVID-19 co-infections in cancer patients and collect longitudinal samples for sequencing throughout infection, such as in Borges et al. (2021). Additionally, we actively avoided linking sequencing information with medical records to protect patient privacy and confidentiality given that we made the sequencing data available in our supplemental materials for this manuscript (Sequence ID which may include county, age, gender on GISAID).

<https://journals.asm.org/doi/pdf/10.1128/msphere.00244-21>
